# The long‐term impact of alveolar ridge preservation with xenograft bone mineral on peri‐implant health after 5 years in function: A retrospective cohort study of 108 patients assessed clinically and radiologically

**DOI:** 10.1002/cre2.583

**Published:** 2022-05-05

**Authors:** Shariel Sayardoust, Wilhelm Norstedt, Furqan A. Shah

**Affiliations:** ^1^ Centre for Oral Health, School of Health and Welfare Jönköping University Jönköping Sweden; ^2^ Department of Periodontology Institute for Postgraduate Dental Education Jönköping Sweden; ^3^ Department of Biomaterials, Sahlgrenska Academy University of Gothenburg Gothenburg Sweden

**Keywords:** alveolar ridge preservation, deproteinized bovine bone, peri‐implantitis, smoking

## Abstract

**Objectives:**

When teeth are lost, dental implants contribute to improved oral function and quality of life. Limitations in dental implant placement arising from poor bone anatomy may be circumvented via alveolar ridge preservation (ARP). The aim is to evaluate the long‐term impact of ARP on peri‐implant health and the relationship with common risk indicators such as smoking and history of periodontitis.

**Materials and Methods:**

One hundred and eight patients were enrolled in this retrospective cohort study with 308 implants. Of these, ∼41% were placed in bone sites that had previously received ARP with deproteinized bovine bone mineral xenograft. Association between baseline variables: ARP, age, gender, number of implants per patient, anatomical site, smoking, and previous history of grade III/IV periodontitis, and outcome variables: mucositis, peri‐implantitis, implant loss, full‐mouth plaque score (FMPS), full‐mouth bleeding score, and marginal bone loss (MBL) was evaluated using both univariate and multivariate models.

**Results:**

After 5 years, the overall survival rate was 93.7%. The occurrence of peri‐implantitis was 21.3% and the extent of MBL was ~2.2 mm. Both peri‐implantitis occurrence and MBL were comparable between ARP^+^ and ARP^−^. Smoking is associated with higher FMPS and MBL.

**Conclusions:**

The findings indicate that peri‐implant health can be maintained around dental implants for up to 5 years in ARP^+^ sites using Bio‐Oss®. Smoking is a major risk indicator for peri‐implantitis, whereas the association between history of periodontitis and the risk of peri‐implantitis, based on this specific, well‐maintained cohort and the specific implants used, remains inconclusive.

## 1 INTRODUCTION

The use of dental implants is widely accepted as a reconstructive treatment modality for tooth replacement. The success of oral implantation depends on overlapping phases of bone formation, subsequent remodeling, and the establishment of a stable interface between bone and the implant surface (Brånemark, 1983; Palmquist et al., 2010; Shah et al., 2019). In this complex process, both local and systemic host factors are of importance (Chrcanovic et al., 2015; Pjetursson & Heimisdottir, 2018; Smeets et al., 2014). A key local factor is the volume of the alveolar ridge, where an implant is to be installed. Clinically, prosthetically driven positioning of the implant is desirable, and to reach that, the physical dimensions of the alveolar bone are important and dictate the dimensions of the implant (Testori et al., 2018). Following tooth loss, there is recession of alveolar bone in both width and height. The rates and patterns of such alveolar bone loss differ between anatomical sites (Tan et al., 2012; Van der Weijden et al., 2009).

Various techniques are used to preserve the alveolar ridge, of which socket filling (or socket preservation) is the most common approach (Avila‐Ortiz et al., 2019; Mardas et al., 2015; Vignoletti et al., 2012). Immediately after tooth extraction, the alveolar socket is filled with a suitable bone graft substitute and sutured, with or without the use of a barrier membrane (Hämmerle et al., 2012). The bone graft substitute provides volumetric stability by acting as a scaffold for bone regeneration (Friedmann et al., 2002). Alveolar ridge preservation (ARP) reduces the risk of suboptimal implant positioning leading to a poor esthetic outcome (Capelli et al., 2013; Chappuis et al., 2000).

Implant success is determined by a number of factors including survival rates, mobility, absence of inflammation and pain, and radiographic bone loss (Misch et al., 2008). During the first year of function, peri‐implant bone loss can be elevated owing to remodeling of the peri‐implant bone (Tarnow et al., 2000). After the initial year, marginal bone loss (MBL) of <0.2 mm/year indicates stable conditions (Albrektsson et al., 1986). The use of ARP techniques has been found to be efficient in many previous studies in reducing resorption of the alveolar ridge, providing the possibility of restoring the site with an implant (Carmagnola et al., 2003; Vignoletti et al., 2012) and reducing the need for bone augmentation at the time of implant placement. However, many important questions around the long‐term effects of ARP with regard to clinical variables, for example, MBL and peri‐implantitis, and the impact of various patient factors such as smoking, and history of periodontitis remain unaddressed.

Here, we undertake a retrospective, clinical and radiological investigation of the impact of ARP using a commercially available deproteinized bovine bone mineral xenograft (Bio‐Oss®) on dental implants after 5 years in function. In addition to clinical features such as MBL, mucositis, the incidence of peri‐implantitis, full‐mouth plaque score (FMPS), and full‐mouth bleeding score (FMBS), and risk factors/indicators, such as smoking, history of periodontitis, and anatomical site (i.e., maxilla or mandible) are considered.

## 2 MATERIALS AND METHODS

### 2.1 Clinical study

The study protocol was approved by the Institutional Review Board at Linköping University, Sweden (EPN‐Dnr 2019‐00554). The study was conducted in accordance with the guidelines of Good Clinical Practice for Trials on Medicinal Products in the European Community, the International Conference on Harmonization (ICH) guideline for Good Clinical Practice, the Declaration of Helsinki, and the STROBE guidelines for clinical studies. Between January 2014 and February 2015, all patients referred to and treated with dental implants at the Department of Periodontology, Institute for Postgraduate Dental Education in Jönköping, Sweden, were consecutively enrolled in the study.

Enrollment was performed on medical records of patients treated with implant prosthesis rehabilitation with 5 years of functional loading, who followed a structured individual maintenance program and supportive periodontal and peri‐implant treatment (SPT).

### 2.2 Inclusion

The selection was performed according to the following inclusion criteria:

(i) Implant treatment between January 2014 and February 2015.
(ii) Absence of risk factors that could affect levels of bone‐related gene expression, including osteoporosis, chronic use of anti‐inflammatory agents, use of antiresorptive agents (e.g., bisphosphonates), or severe metabolic diseases such as diabetes mellitus.
(iii) Implants included only Straumann® Standard Plus SLA® implants (Institut Straumann AG, Basel, Switzerland).
(iv) ARP with only xenograft (Bio‐Oss®; Geistlich Pharma AG, Wolhusen, Switzerland).
(v) No antibiotics are used within 3 months before tooth extraction or until 1 month after implant placement.

### 2.3 Exclusion

Exclusion criteria were systemic diseases, poor oral hygiene at baseline, uncontrolled periodontal disease, severe intermaxillary skeletal discrepancy, previous radiotherapy to the head and neck region for malignancy, and former smokers.

### 2.4 Enrollment and patient demographics

A total of 108 consecutive patients were found suitable for further investigation (Table 1). All patients included provided oral and written consent for participation in the present study. Forty‐seven out of 108 patients underwent ARP with Bio‐Oss® (∼25 mg for 1–2 alveoli), ∼4–7 months before implant placement.

**Table 1:** Demographic information

	Patient level			Implant level		
	All (*n* = 108)	ARP^+^ (*n* = 47)	ARP^−^ *(n* = 61)	All (*n* = 309)	ARP^+^ (*n* = 127)	ARP^−^ (*n* = 182)
Age (years)^a^	61.2 ± 14.7	60.8 ± 16.2	61.6 ± 13.7	63.7 ± 12.3	64.5 ± 13.5	63.1 ± 11.3
Gender (M:F)	1	1.14	0.91	0.93	0.87	0.98
Implants (per patient)^a^	2.86 ± 1.8	2.7 ± 1.9	2.98 ± 1.8	3.98 ± 1.74	3.96 ± 1.84	4.0 ± 1.68
Site (Max:Mand)	1.25	1.76	0.97	0.86	1.23	0.67
Smoking (%)	29.6	27.7	31.1	29.5	26.8	31.3
Periodontitis (%)	35.2	34	36.1	37.5	35.43	39.01

*Note*: Gender: Ratio of M to F participants. Site: Ratio of Max to Mand cases. Smoking: Percentage incidence of current smoking history. Periodontitis: Percentage incidence of the previous history of stage III or IV periodontitis.

Abbreviations: ARP, alveolar ridge preservation; F, female; M, male; Mand, mandible; Max, maxilla.

^a^ Mean value ± standard deviation.

This retrospective cohort study included 108 systemically healthy patients (54 males and 54 females) aged between 26 and 87 years (Table 1). ARP was performed in 47 patients (designated as ARP^+^), 6 months before placement of 127 implants (2.7 ± 1.9 implants per patient). The remaining 61 patients received 182 implants (2.98 ± 1.8 implants per patient) without ARP (designated as ARP^−^). A total of 143 implants were placed in the maxilla and 166 implants in the mandible. Out of the 108 patients, 38 had previously received treatment for stage III or IV periodontitis, in accordance with the 2017 World Workshop on the Classification of Periodontal and Peri‐Implant Diseases and Conditions (Papapanou et al., 2018). At the time of implant placement, all patients were periodontally healthy. Ninety‐six out of the 308 implant sites were single‐rooted. Thirty‐eight percent of the extractions were due to periodontal disease.

### 2.5 Implant treatment

All surgeries were performed by three qualified, specialist periodontists at the Department of Periodontology, Institute for Postgraduate Dental Education. All the steps of the treatments were thoroughly documented in the medical records for each patient. Before dental implant surgery, all patients were required to attain an FMPS <20%, in addition to the absence of pocket depth of >5 mm with bleeding on probing. To be treated with ARP the patients had <1 mm thickness of buccal bone remaining after atraumatic tooth extraction. The ARP was performed using Bio‐Oss®. Particle morphology, microstructure, and chemical composition were characterized using optical microscopy, backscattered electron scanning electron microscopy, energy dispersive X‐ray spectroscopy, micro‐Raman spectroscopy, and X‐ray diffraction (see Supporting Information). Before application to the extraction alveoli, Bio‐Oss® is mixed with autogenous blood. The protocol recommended by the manufacturer was used. No membrane was used in combination with the ARP. Single sutures (Vicryl™; Ethicon) were applied. An average healing time of 20 ± 2 weeks was allowed before proceeding with implant treatment. No temporary prosthesis was used.

Standard drilling sequences were followed, as suggested by the implant manufacturer. After a healing time of 8–12 weeks, a screw‐retained fixed prosthesis was mounted. which in most cases was a multiunit prosthesis. Only 36 patients received single‐unit prostheses. The distribution of single‐ and multiunit prostheses was similar for ARP^+^ and ARP^−^. All patients were offered a structured maintenance program with a regular recall interval of 5–6 months.

### 2.6 Structured recall and maintenance program

After surgery, all patients followed an individually structured maintenance program. The SPT consisted of follow‐up visits and oral hygiene professional sessions, with motivational dialogs, instructions for mechanical removal of bacterial plaque, and daily oral hygiene procedures. Follow‐up visits were scheduled for 1, 3, 6, and 12 months during the first postoperative year and individualized to 2–5 visits annually thereafter. During each recall, the patients underwent a complete clinical assessment of peri‐implant soft tissue conditions.

### 2.7 Clinical assessments

The FMPS and FMBS were assessed (Ainamo & Bay, 1975). All periodontal and peri‐implant assessments were performed on the four sides (buccal, lingual/palatal, mesial, and distal) by three dental hygienists who were unaware of the treatment(s) provided. The same clinical examinations were carried out as routine examinations after 5 years. The pocket probing depth was measured (Supporting Information: Figure S1). Implant survival and MBL were analyzed. Diagnosis for mucositis and peri‐implantitis was made in accordance with the 2017 World Workshop on the Classification of Periodontal and Peri‐Implant Diseases and Conditions (Berglundh et al., 2018).

### 2.8 Radiological assessment

All patients were radiologically examined at a core facility with experienced specialists in dental radiology. Digital intraoral radiographs were obtained using a long‐cone paralleling technique at baseline (time point when the fixed prosthesis was mounted) and the 5‐year follow‐up. MBL was measured by one examiner (Shariel Sayardoust) blinded to implant group allocation. The distance between a reference point (implant‐abutment junction or implant head‐prosthetic construction) and the marginal bone level at the mesial and distal sides of each implant was recorded. Of these, the greater value was considered. In fewer than 5% of instances, due to radiographic artifacts or anatomy, only one of the sides could be accurately measured. The built‐in measurement function in the image archiving and communication system corrected for the magnification. Analysis of intraexaminer consistency (intraclass correlation coefficient 0.92) was carried out 1 month after the measurements were obtained.

### 2.9 Primary outcome

*Peri‐implantitis* is defined as the presence of bleeding and/or suppuration on gentle probing with probing depths ≥6 mm and crestal bone levels ≥3 mm apical to the most coronal portion of the intraosseous part of the implant (Berglundh et al., 2018).

### 2.10 Secondary outcomes

*Mucositis* is defined as the presence of bleeding and/or suppuration on gentle probing with or without increased probing depth compared to previous examinations. In addition to the absence of bone loss beyond the crestal bone level, changes result from initial bone remodeling (Berglundh et al., 2018).

*MBL* is defined as the vertical distance from the restoration margin to the most coronal level of implant–bone contact at the mesial and distal aspects of each implant

*FMPS*, dichotomous registration (O'Leary et al., 1972).

*FMBS*, dichotomous registration (Ainamo & Bay, 1975).

*Implant loss* is defined as the absence in the mouth and functioning at the end of the 5‐year follow‐up.

### 2.11 Statistical analysis

Logistic regression analysis was performed for binary outcomes as dependent variables, that is, mucositis, peri‐implantitis, and implant loss. Linear regression analysis was performed for continuous outcomes as dependent variables, that is, FMPS, FMBS, and MBL. In both cases, the independent variables were ARP, age, gender, number of implants per patient, anatomical site (maxilla or mandible), smoking, and previous history of stage III/IV periodontitis. Both univariate and multivariate models were used, considering the individual patient as the statistical unit. The results of the multilevel modeling were consistent with the patient‐based analysis. The outcomes are presented at the patient level. In the multivariate analysis, ARP was included together with all other baseline variables that had a *p* < .15 in the univariate analysis. Odds ratio (OR, logistic regression) and *β*‐coefficient (linear regression), together with a 95% confidence interval are presented, and *p* < .05 was considered significant.

## 3 RESULTS

### 3.1 Patient demographics

This retrospective cohort study included 108 systemically healthy patients (54 males and 54 females) aged between 26 and 87 years (Table 1). ARP was performed in 47 patients (designated as ARP^+^), 6 months before placement of 127 implants (2.7 ± 1.9 implants per patient). The remaining 61 patients received 182 implants (2.98 ± 1.8 implants per patient) without ARP (designated as ARP^–^). A total of 143 implants were placed in the maxilla and 166 implants in the mandible. Out of the 108 patients, 38 had previously received treatment for stage III or IV periodontitis, in accordance with the 2017 World Workshop on the Classification of Periodontal and Peri‐Implant Diseases and Conditions (Papapanou et al., 2018). At the time of implant placement, all patients were periodontally healthy.

### 3.2 Clinical outcomes after 5 years

**Figure 1:**
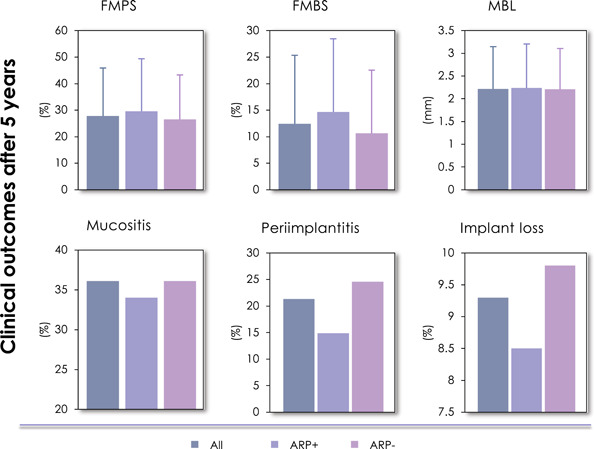
Clinical outcomes after 5 years. ARP, alveolar ridge preservation; FMBS, full‐mouth bleeding score; FMPS, full‐mouth plaque score; MBL, marginal bone loss.

A total of 13 (out of 308) implants were lost in 10 (out of 108) patients, indicating a survival rate of 93.7% on the patient level. 36.1% of the patients had mucositis and 21.3% of the patients exhibited peri‐implantitis (Figure 1). This cohort of patients had after 5 years of implants in function FMPS and FMBS of 27.7 ± 18.1% and 12.4 ± 12.9%, respectively, while MBL was ∼2.2 mm on the patient level. No significant differences were noted between ARP^+^ and ARP^–^.

### 3.3 Mucositis, peri‐implantitis, and implant loss

**Figure 2:**
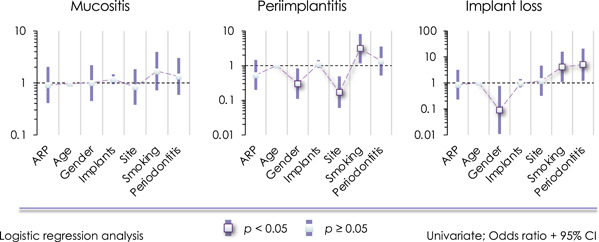
Univariate logistic regression analysis. ARP, alveolar ridge preservation; CI, confidence interval.

The univariate (Figure 2) and multivariate (Supporting Information: Tables S1 and S2) analyses indicate that the tendency for implant loss is lower in men, while a previous history of periodontitis substantially increases the potential for implant loss. No relationship exists between ARP and the occurrence of mucositis, peri‐implantitis, or implant loss. However, peri‐implantitis appears to be less likely in the maxilla than in the mandible. Moreover, the likelihood of peri‐implantitis is lower in males compared to females and in the maxilla. The likelihood of developing mucositis is higher with a previous history of periodontitis, a greater number of implants per patient, and increases with advancing age.

### 3.4 FMPS, FMBS, and MBL

**Figure 3:**
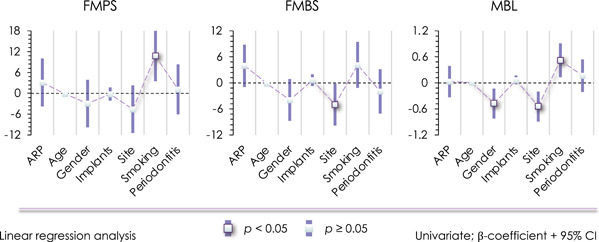
Univariate linear regression analysis. ARP, alveolar ridge preservation; CI, confidence interval; FMBS, full‐mouth bleeding score; FMPS, full‐mouth plaque score; MBL, marginal bone loss.

The univariate (Figure 3) and multivariate (Supporting Information: Tables S3 and S4) analyses indicate that MBL tends to be lower in the maxilla compared to the mandible. No relationship exists between ARP and FMPS, FMBS, and MBL.

### 3.5 Impact of smoking on mucositis, peri‐implantitis, implant loss, FMPS, FMBS, and MBL

The univariate (Figure 2) and multivariate analyses (Supporting Information: Tables S1 and S2) indicate that smoking greatly increases the occurrence of peri‐implantitis (Figure 3), and smoking is associated with higher FMPS and MBL.

### 3.6 Morphological and chemical characterization

**Figure 4:**
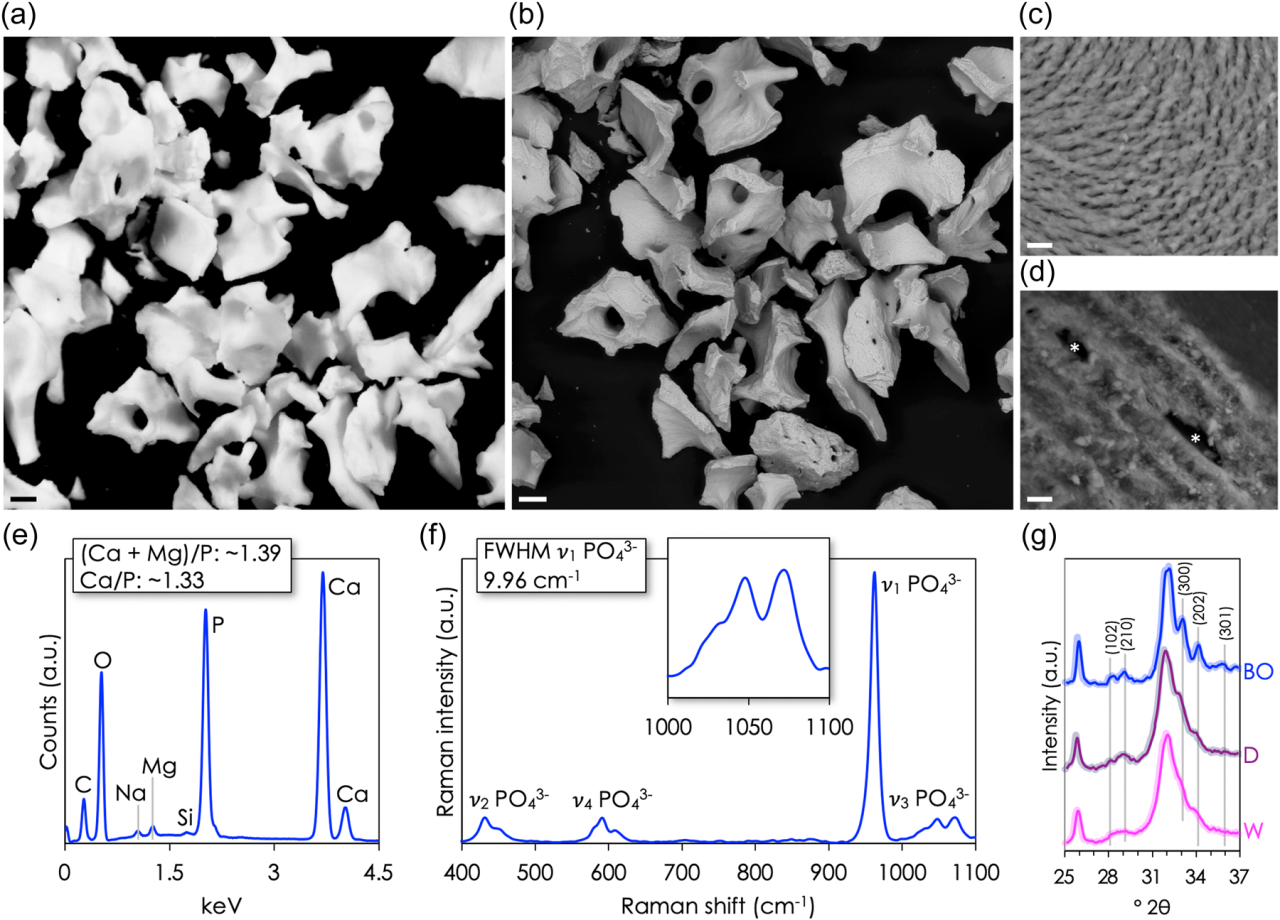
(a) Optical image. (b–d) Backscattered electron scanning electron microscopy. (c) Typical appearance of bone mineral arranged into micrometer‐sized, rope‐like bundles. (d) The lamellar structure and empty osteocyte lacunae (*) are seen in cross‐section. (e) Energy dispersive X‐ray spectroscopy. (f)  Raman spectroscopy. (g) X‐ray diffraction. Bovine bone, whole (W; magenta); bovine bone, NaOCl deproteinized (D; purple); and Bio‐Oss® (BO; blue). Scale bars in (a,b) = 250 µm and in (c,d) = 5 µm. FWHM, full‐width at half‐maximum.

Bio‐Oss® granules, ∼250 µm to ∼1 mm in size, exhibit surface microstructure similar to deproteinized bone (Figure 4), where mineralized collagen fibrils assemble into a network of twisted rope‐like bundles (Shah, Ruscsák, et al., 2020; Shah, Zanghellini, et al., 2016). Fractured surfaces reveal a lamellar pattern. Elemental analysis reveals a Ca/P ratio of ∼1.33 and Mg enrichment. Very low amounts of Si are also detected. Raman spectroscopy demonstrates the absence of the organic constituents of the extracellular matrix (i.e., collagen). The v_1_ CO_3_^2^^−^ band is weak, while the CO_3_^2^^−^ content (ν_1_ CO_3_^2^^−^/v_1_ PO_4_^3^^−^ intensity ratio) is 0.096. Furthermore, a distinct peak attributable to the v_3_ PO_4_^3^^−^ band is evident at 1047 cm^−1^. Taken as the inverse full‐width at half‐maximum of the v_1_ PO_4_^3^^−^ band, the mineral crystallinity of Bio‐Oss® is ∼0.1. X‐ray diffraction reveals higher mineral crystallinity of Bio‐Oss® compared to whole bovine bone and deproteinized bovine bone, where in the 32–35°2θ region, Bragg peaks attributable to the (102), (210), (300), (202), and (301) reflections are clearly resolved.

## 4 DISCUSSION

Evaluation of the long‐term performance of dental implants is an important clinical aspect. The most common biological complications associated with dental implants are mucositis and peri‐implantitis. Common to both mucositis and peri‐implantitis is the presence of an inflammatory lesion. However, the difference between them is that the latter indicates bone loss adjacent to the implant at rates exceeding those of bone turnover (Araújo & Lindhe, 2009; Jepsen et al., 2015; Sanz & Chapple, 2012), and evident radiographically, while mucositis describes only bleeding on gentle probing, according to the 2017 World Workshop on the Classification of Periodontal and Peri‐Implant Diseases and Conditions (Berglundh et al., 2018).

Measurement of MBL around dental implants, in combination with other parameters such as FMBS, is considered to be a valuable indicator of biological complications. Therefore, in this retrospective study, we investigate mucositis, peri‐implantitis, FMPS, FMBS, MBL, and implant loss to assess the long‐term performance of dental implants after 5 years in function.

ARP is considered a beneficial approach in mitigating the dimensional loss of the alveolar ridge, enabling the possibility of restoring the site with an implant (Carmagnola et al., 2003; Vignoletti et al., 2012) and thereby eliminating the need for bone augmentation at the time of implant placement. Studies comparing the outcome of implants placed in the augmented bone and in native bone are not consistent, hence different techniques, different surgical methods, different biomaterials, different implant surfaces, healing times, and so forth, are used. Two recent systematic reviews have addressed the issue of ARP and its effect on biological complications and implant failure. It is concluded by both that there is great heterogeneity in the published literature in terms of bone graft substitutes used and surgical techniques (Ramanauskaite et al., 2019; Salvi et al., 2018). Salvi et al. (2018) report no differences in the outcome between implants placed in ARP^+^ sites, using various materials, when compared to ARP^−^ sites with regard to complications around dental implants (Salvi et al., 2018). On the other hand, the findings of Ramanauskaite et al. (2019) indicate high survival rates and lower MBL in ARP^+^ sites compared to ARP^−^ sites. Although we report comparable MBL values for ARP^+^ and ARP^−^ sites at 5‐year follow‐up, it must be noted that the patients designated to receive ARP are already at a disadvantage, with a minimal to nonexistent buccal bone after tooth extraction.

There is a large variation of biomaterials (i.e., bone graft substitutes) and techniques associated with ARP procedures (Avila‐Ortiz et al., 2019; Mardas et al., 2015; Vignoletti et al., 2012). Xenografts are commonly used graft materials in oral surgery due to their osteoconductive properties, volume stability, and most importantly no donor site morbidity (Haugen et al., 2019). Bio‐Oss® is one such xenograft material based on deproteinized bovine bone. Not only is the microstructure of bovine bone similar to that of human bone, but the porous design also provides a scaffold for bone ingrowth. Nevertheless, histological studies of Bio‐Oss® reveal slow resorption (Orsini et al., 2007; Schlegel & Donath, 1998), which is related to the physicochemical characteristics of Bio‐Oss®. More specifically, the CO_3_^2^^−^ content (ν_1_ CO_3_^2^^−^/ν_1_ PO_4_^3^^−^ intensity ratio) is ∼44%–52% lower than values typically reported for whole bone (Shah, Ruscsák, et al., 2020; Shah, Snis, et al., 2016), indicating significant decarboxylation, while the mineral crystallinity is ∼71.5% higher than whole bovine bone (Shah, 2020).

A major strength of the present study is that in addition to only one surgical technique (i.e., a flapped approach) without the use of a guided bone regeneration (GBR) membrane, the same bone graft substitute and the same implant design are used, thereby providing consistency and reproducibility. At the same time, the fact that patients could not have been randomized into ARP^+^ and ARP^–^ groups may be considered a limitation of the present work. Techniques commonly used for ARP include open flap or flapless, with or without GBR (Avila‐Ortiz et al., 2019). Although a flapped approach provides easy access to the surgical site, there is currently little consensus regarding the efficacy of using a flapped approach (Avila‐Ortiz et al., 2014; Vignoletti et al., 2012) over a flapless approach (Fickl et al., 2008), or any particular advantage between the two techniques (Araújo & Lindhe, 2009). More recently, an active role of GBR membranes has been demonstrated in promoting regeneration within an osseous defect, rather than functioning simply as a passive barrier (Elgali et al., 2017).

Studies on the performance and effects of ARP in treatment with implants are ambiguous. In agreement with our findings, a recent study reports a similar outcome for implants placed in nonmolar ARP^–^ and ARP^+^ sites after 1 year (Lim et al., 2020). The same study also did not find any differences in the esthetic outcome and patient discomfort. Similarly, in the esthetic zone (anterior region) of the maxilla, we did not detect any differences between the outcome of implants placed at ARP^–^ and ARP^+^ sites after 1 year (Zuiderveld et al., 2019). Besides ARP^+^ sites presenting a lower need for grafting at the time of implant placement and accommodating for larger diameter implants compared to ARP^–^ sites, reveal no difference(s) in the overall outcome of ARP^–^ and ARP^+^ after 3 years (Barone et al., 2012). Indeed, this outcome is neither unusual nor unexpected and has been reported frequently.

In the literature, smoking is often discussed as a risk indicator for biological complications around dental implants. Typically, studies evaluating the impact of smoking take into consideration only MBL and implant loss (Chrcanovic et al., 2016; De Bruyn & Collaert, 1994; Heitz‐Mayfield, 2008; Heitz‐Mayfield & Huynh‐Ba, 2009; Sayardoust et al., 2013). Although it has recently been suggested that the association between smoking and peri‐implantitis (i.e., smoking being a risk factor for peri‐implantitis) is inconclusive (Schwarz et al., 2018), we report approximately three times higher odds of peri‐implantitis in smokers compared to nonsmokers. Furthermore, implant loss strongly correlates with smoking habits.

History of periodontitis also contributes significantly to implant loss (OR: 5.04). In contrast to common knowledge and published literature indicating that a history of periodontitis correlates with peri‐implantitis (Heitz‐Mayfield, 2008; Heitz‐Mayfield & Huynh‐Ba, 2009; Karoussis et al., 2003), our findings do not reveal an elevated risk of peri‐implantitis or mucositis in this cohort of patients with a history of periodontitis, which we attribute to the structured maintenance program offered. This outcome is in agreement with the notion of a protective effect of periodontal/peri‐implant maintenance in patients with a previous history of periodontitis (Roccuzzo et al., 2018), as it is evident that a defined and structured maintenance program following placement of a dental implant lowers the incidence of peri‐implantitis at 5–7 year follow‐up by 59%–76% (Costa et al., 2012; Hu et al., 2020). A systematic review article investigating the role of a structured maintenance program found sufficient evidence to suggest a minimum recall period of 5–6 months (Monje et al., 2016). The role of supportive care also extends to implants treated for peri‐implantitis, where medium to long‐term follow‐up correlates with a high implant survival rate (Roccuzzo et al., 2018).

Within the limitation(s) of this retrospective study, it is concluded that ARP can be used to maintain the marginal bone level around implants and that at 5‐year follow‐up, both hard tissue and soft tissue conditions may be expected to be generally comparable between ARP^−^ and ARP^+^ sites treated with deproteinized bovine bone (e.g., Bio‐Oss®) or other xenograft materials similar in terms of physical and chemical characteristics. It is also revealed that in this specific well‐maintained cohort, a history of periodontitis does not affect the risk of peri‐implantitis around the specific implants studied.

## AUTHOR CONTRIBUTIONS

*Substantial contributions to the conception and design of the work; and the acquisition, analysis, and interpretation of data for the work; drafting the work and revising it critically for important intellectual content; final approval of the version to be published; and agreement to be accountable for all aspects of the work in ensuring that questions related to the accuracy and integrity of any part of the work are appropriately investigated and resolved*: Shariel Sayardoust. *Contributions to the acquisition, analysis, and interpretation of data for the work; drafting the work; final approval of the version to be published; and agreement to be accountable for all aspects of the work in ensuring that questions related to the accuracy and integrity of any part of the work are appropriately investigated and resolved*: Wilhelm Norstedt. *Substantial contributions to the conception and design of the work and interpretation of data for the work; revising it critically for important intellectual content; final approval of the version to be published; and agreement to be accountable for all aspects of the work in ensuring that questions related to the accuracy and integrity of any part of the work are appropriately investigated and resolved*: Furqan A. Shah.

## CONFLICTS OF INTEREST

The authors declare no conflicts of interest.

## Supporting information

Supporting information.

## Data Availability

The data that support the findings of this study are available from the corresponding author upon reasonable request.
