# Supplementary material for: The long‐term impact of alveolar ridge preservation with xenograft bone mineral on peri‐implant health after 5 years in function: A retrospective cohort study of 108 patients assessed clinically and radiologically
Source: Clin Exp Dent Res. 2022 May 5;8(3):640–9. doi: 10.1002/cre2.583 (PMC9209797; doi:10.1002/cre2.583)
Supplement: Supplementary file 1 — Supporting information. [file CRE2-8-640-s001.docx]

**SUPPORTING INFORMATION**

**The long-term impact of alveolar ridge preservation with xenograft bone mineral on peri-implant health after 5 years in function: A retrospective cohort study of 108 patients assessed clinically and radiologically**

**Running title:** Alveolar ridge preservation with xenograft

Shariel Sayardoust^1, 2, 3^ ([shariel.sayardoust@ju.se](mailto:shariel.sayardoust@ju.se))

Wilhelm Norstedt ^2^ ([wilhelm.norstedt@rjl.se](mailto:wilhelm.norstedt@rjl.se))

Furqan A. Shah^3^ ([furqan.ali.shah@biomaterials.gu.se](mailto:furqan.ali.shah@biomaterials.gu.se))

^1^Centre for Oral Health, School of Health and Welfare, Jönköping University, Jönköping, Sweden

^2^Department of Periodontology, Institute for Postgraduate Dental Education, Jönköping, Sweden

^3^Department of Biomaterials, Sahlgrenska Academy, University of Gothenburg, Gothenburg, Sweden

******Corresponding author:***

Shariel Sayardoust

ORCID: 0000-0003-3738-1217

Email: [shariel.sayardoust@ju.se](mailto:shariel.sayardoust@ju.se)

Tel: +46 73 656 4648

**Deproteinized bovine bone – morphology, microstructure and chemical composition**

The xenograft (Bio-Oss®, Geistlich Pharma AG, Wolhusen, Switzerland) was used as the bone graft substitute material, which is obtained by complete removal of organic and cellular components of the extracellular matrix of bovine bone. Particle morphology was examined by optical microscopy (Nikon SMZ1500 stereomicroscope equipped with an HR Plan Apo 1X objective lens) and scanning electron microscopy (SEM, Quanta 200 environmental SEM, FEI Company, The Netherlands) in the backscattered electron mode (BSE) operated at 20 kV accelerating voltage, 1 Torr water vapor pressure, and 10 mm working distance. Elemental analysis was performed using energy dispersive X-ray spectroscopy (INCA EDX system, Oxford Instruments GmbH, Wiesbaden, Germany).

Micro-Raman spectroscopy was performed using a confocal Raman microscope (Renishaw inVia^TM^ Qontor®) equipped with a 633 nm laser and LiveTrack^TM^ focus-tracking technology. The laser was focused down on to the deproteinised bovine bone particle surface using a ×100 objective. Spectra were collected in the ~400–1,100 cm^-1^ spectral range using a Peltier-cooled charge-coupled device deep depletion near-infrared enhanced detector behind a 2,400 g mm^-1^ grating. Background fluorescence subtraction and cosmic ray removal were performed in Renishaw WiRE 5.4 software.

For direct comparison of mineral crystallinity, X-ray diffraction measurements were obtained from Bio-Oss®, whole bovine bone, and deproteinised bovine bone (5% NaOCl (14h at 4 °C), using a Bruker D8 Advance X-ray diffractometer. Fresh frozen bovine bone was obtained from a local slaughterhouse, stored in Hank’s balanced salt solution at 4 °C until use, manually crushed into < 500 µm sized particles, with or without additional processing, followed by dehydration in a graded series of ethanol (50–100%).

**
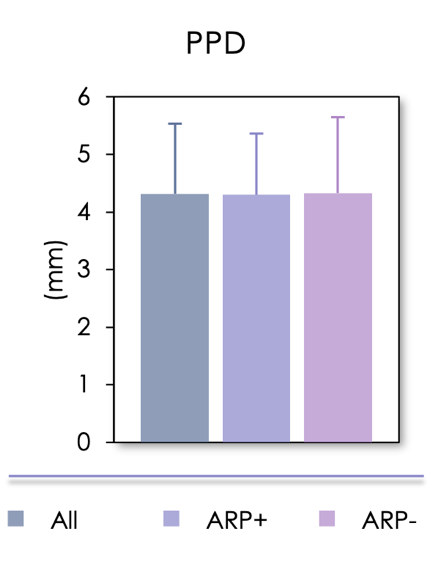
**

**Fig. S1. Probing pocket depth (PPD)**

**Supplementary Table S1. Univariate logistic regression**

|  | Mucositis | | Periimplantitis | | Implant loss | |
| --- | --- | --- | --- | --- | --- | --- |
|  | **OR**  **(95% CI)** | ***p*** | **OR**  **(95% CI)** | ***p*** | **OR**  **(95% CI)** | ***p*** |
| ARP | 0.91  (0.41, 2.03) | >0.30 | 0.54  (0.20, 1.45) | 0.22 | 0.85  (0.23, 3.21) | >0.30 |
| Age | 0.97  (0.95, 1.01) | 0.055 | 1.02  (0.98, 1.06) | 0.29 | 1.00  (0.96, 1.05) | >0.30 |
| Gender | 1.00  (0.45, 2.20) | >0.30 | **0.30**  **(0.11, 0.83)** | **0.021** | **0.09**  **(0.01, 0.77)** | **0.028** |
| Implants | 1.18  (0.95, 1.47) | 0.14 | 1.11  (0.86, 1.44) | >0.30 | 0.98  (0.68, 1.41) | >0.30 |
| Site | 0.83  (0.38, 1.84) | >0.30 | **0.17**  **(0.06, 0.49)** | **0.0012** | 1.22  (0.32, 4.60) | >0.30 |
| Smoking | 1.69  (0.72, 3.94) | 0.23 | **3.09**  **(1.17, 8.16)** | **0.022** | **4.15**  **(1.09, 15.90)** | **0.038** |
| Periodontitis | 1.33  (0.59, 3.02) | >0.30 | 1.37  (0.52, 3.55) | >0.30 | **5.04**  **(1.22, 20.82)** | **0.025** |

*Age*: OR per year. *Implants (per patient)*: OR per implant

**Supplementary Table S2. Multivariate logistic regression**

|  | Mucositis | | Periimplantitis | | Implant loss | |
| --- | --- | --- | --- | --- | --- | --- |
|  | **OR**  **(95% CI)** | ***p*** | **OR**  **(95% CI)** | ***p*** | **OR**  **(95% CI)** | ***p*** |
| ARP | 0.95  (0.41, 2.19) | >0.30 | 0.66  (0.22, 1.94) | >0.30 | 0.59  (0.11, 3.18) | >0.30 |
| Age | **0.96**  **(0.94, 0.99)** | **0.015** | - | - | - | - |
| Gender |  |  | 0.46  (0.15, 1.41) | 0.18 | **0.033**  **(0.0030, 0.36)** | **0.0050** |
| Implants | **1.30**  **(1.02, 1.65)** | **0.035** | - | - | - | - |
| Site | - | - | **0.22**  **(0.071, 0.69)** | **0.0091** | - | - |
| Smoking | - | - | 2.29  (0.79, 6.48) | 0.12 | 1.91  (0.39, 9.46) | >0.30 |
| Periodontitis | - | - | - | - | **16.16**  **(2.81, 92.94)** | **0.0018** |

*Age*: OR per year. *Implants (per patient)*: OR per implant

**Supplementary Table S3. Univariate linear regression**

|  | FMPS | | FMBS | | MBL | |
| --- | --- | --- | --- | --- | --- | --- |
|  | **β-coefficient**  **(95% CI)** | ***p*** | **β-coefficient**  **(95% CI)** | ***p*** | **β-coefficient**  **(95% CI)** | ***p*** |
| ARP | 3.11  (-3.79, 10.02) | >0.30 | 3.94  (-0.94, 8.81) | 0.11 | 0.030  (-0.33, 0.39) | >0.30 |
| Age | -0.20  (-0.43, 0.033) | 0.093 | -0.11  (-0.28, 0.055) | 0.19 | 0.0086  (-0.0042, 0.021) | 0.19 |
| Gender | -2.94  (-9.80, 3.91) | >0.30 | -3.93  (-8.75, 0.90) | 0.11 | **-0.47**  **(-0.82, -0.13)** | **0.0076** |
| Implants | -0.22  (-2.13, 1.70) | >0.30 | 0.64  (-0.71, 2.00) | >0.30 | 0.068  (-0.031, 0.17) | 0.18 |
| Site | -4.57  (-11.43, 2.29) | 0.19 | **-4.99**  **(-9.82, -0.17)** | **0.043** | **-0.54**  **(-0.89, -0.20)** | **0.0022** |
| Smoking | **10.73**  **(3.48, 17.97)** | **0.0037** | 4.16  (-1.13, 9.45) | 0.12 | **0.52**  **(0.13, 0.91)** | **0.0083** |
| Periodontitis | 1.15  (-6.05, 8.34) | >0.30 | -1.96  (-7.07, 3.14) | >0.30 | 0.17  (-0.21, 0.54) | >0.30 |

**Supplementary Table S4. Multivariate linear regression**

|  | FMPS | | FMBS | | MBL | |
| --- | --- | --- | --- | --- | --- | --- |
|  | **β-coefficient**  **(95% CI)** | ***p*** | **β-coefficient**  **(95% CI)** | ***p*** | **β-coefficient**  **(95% CI)** | ***p*** |
| ARP | 3.34  (-3.30, 9.99) | >0.30 | **4.86**  **(0.045, 9.67)** | **0.048** | 0.12  (-0.22, 0.46) | >0.30 |
| Age | -0.17  (-0.39, 0.058) | 0.15 | - | - | - | - |
| Gender | - | - | -2.43  (-7.39, 2.53) | >0.30 | -0.31  (-0.66, 0.045) | 0.088 |
| Implants | - | - | - | - | - | - |
| Site | - | - | -4.63  (-9.62, 0.36) | 0.069 | **-0.41**  **(-0.77, -0.057)** | **0.023** |
| Smoking | **10.40**  **(3.16, 17.63)** | **0.0049** | 3.05  (-2.26, 8.35) | 0.26 | **0.39**  **(0.012, 0.77)** | **0.043** |
| Periodontitis | - |  | - | - | - | - |
